# The German Food Bank System and Its Users—A Cross-Sectional Study

**DOI:** 10.3390/ijerph15071485

**Published:** 2018-07-13

**Authors:** Anja Simmet, Peter Tinnemann, Nanette Stroebele-Benschop

**Affiliations:** 1 Institute of Nutritional Medicine, University of Hohenheim, 70593 Stuttgart, Germany; N.Stroebele@uni-hohenheim.de; 2 Institute for Social Medicine, Epidemiology, and Health Economics at the Charité University Medical Center Berlin, 10117 Berlin, Germany; peter.tinnemann@charite.de

**Keywords:** food bank, food insecurity, welfare recipients, poverty, food supply, food aid

## Abstract

Although food banks are a well-known resource for low-income people struggling to meet their food needs, they have rarely been investigated on a large scale. This study aims to contribute to the actual debate about the potential and limitations of food banks to decrease the prevalence of food insecurity by providing a representative picture of the German food bank system and its users. Publicly accessible data were used to map residents, public welfare recipients, and food banks. In addition, a comprehensive survey was distributed to all 934 “Tafel” food banks. The results show that nearly all residents and welfare recipients have access to at least one food bank located in the districts in which they reside. Differences in the density of food banks exist between eastern and western Germany. Food banks provide mainly healthy fresh food, but they heavily rely on food donations from local retailers and on volunteer labor. Although changes in the number of user households by income seem to mirror trends in the number of welfare recipients, food bank users appear to represent only a fraction of the food-insecure population in Germany. Food banks might have the potential to improve users’ diet and food security, but they are not able to reach all food-insecure residents in Germany.

## 1. Introduction

Over the last decades, food banks have become a critical food source for people with lowincome in many high-income countries including the USA [1], Canada [2], Australia [3], and in several European countries [4,5,6,7]. Although operation and organization of food banks differ widely between and even within countries, food banks are generally operated by charitable organizations that collect, store, and distribute food donated by retailers, the food industry, and farmers to needy people or to other charitable organizations [8].

Despite the differences in the social security systems across high-income countries, there seem to be commonalities in the characterization of food bank users. For instance, food banks initially aimed to provide temporary emergency assistance to people with financial hardships, whereas users today tend to visit food banks regularly for many years [2,6].

In Germany, the food bank system is called “Tafel” (table) and was initiated in 1993 to help homeless people in Berlin. To date, over 930 local branches of the Tafel food banks have been established throughout the country and they no longer limit assistance to homeless people but assist people with a very low or no income. Food banks usually apply eligibility criteria such as an income at or below the federal unemployment pay (Arbeitslosengeld II) and residence in the coverage area of the food bank [9,10], but in contrast to food banks in other countries such as in the UK [11] and the Netherlands [12], there is no referral system and social workers do not need to be involved. Most food banks in Germany collaborate as members under the umbrella of the federal association “Tafel Deutschland” (Table Germany). Member food banks are solely financed by donations and receive no public financing [13]. As defined by the European Food Banks Federation, a member of which Tafel Deutschland just recently became [14], food banks serve as charitable organizations that help the poor. In this paper we use the expression “food bank” to describe all Tafel entities.

One of the few studies undertaken among food bank users in Germany showed that 70% of over 1000 participating food bank users in three German cities suffered from food insecurity [15]. Thus, food bank users in Germany seem to be less food insecure compared with food bank users in the USA [1], Canada [16], and the Netherlands [7], but they are seven to ten times more often food insecure than the general population [17]. In accordance with studies conducted in other countries [4,6,7], the study found a high prevalence of overweightness and obesity among food bank users (around 68%) [15], in particular among users born outside of Germany [18]. In another study, Depa et al. also revealed that the proportion of people who reported consuming fruit at least daily was lower among the 276 food bank users enrolled than among the representative population with a low socioeconomic status [6]. In addition, around 60% of participating food bank users reported suffering from at least one chronic disease including hypertension, diabetes, or mental illnesses [6]. In summary and in line with international results [4,7,19,20], food bank users in Germany seem to be a very vulnerable population group at high risk of having unfavorable health behaviors and health conditions.

Food banks may serve as an important civil societal resource through their low-threshold services and their nationwide structure. Although the German welfare system is considered more generous compared with those of other countries [21], the Tafel food bank system is the only nationwide immediate food assistance for people struggling to meet their food needs. However, in Germany there is no legal claim to a food bank’s assistance and the nationwide distribution of food banks in relation to the general population and welfare recipients is unknown. Information on food banks’ activities as well as user characteristics are missing on the national level. Studies on the food bank movement have only included samples from few regions or cities [6,9,15,22,23]. The evolution of food banks and rough estimates of the number of users have been illustrated through reports by Tafel Deutschland [24], but a scientific approach to characterize and describe the food bank system and its users on a national level is still missing.

This study aims to provide a representative overview of the German food bank system and its users by

- presenting the coverage rate of food banks in relation to the proportion of welfare recipients in German districts;
- illustrating food banks’ structures, activities, and resources;
- counting and characterizing food bank users by source of household income;
- investigating the association of the number of food bank users and food bank resources and the proportion of welfare recipients in the district the food bank is located in, as well as between the main challenges of food banks and resources and demands of the food banks.

To do this, an explorative cross-sectional study was conducted. Freely accessible data of Tafel Deutschland and the Federal Office of Statistics were used and a comprehensive survey was distributed among all food banks associated with Tafel Deutschland. Illustrating the resources and demands of the food bank system will help to evaluate the potential of food banks to improve the users’ food security level and dietary quality.

## 2. Materials and Methods

The study area included the entire country of Germany consisting of 432 districts and district-free cities (counties, “Landkreise, kreisfreie Städte”) and of 11,437 municipalities (“Gemeinden”) [25]. Tafel Deutschland provided a current list of all registered member food banks [26].

The cross-sectional survey took place from 13 September until 5 December 2017. All food banks received an email containing information about the study, its aims, the voluntary basis of participation, and data protection. The email included a link to a comprehensive online survey, which took approximately 60 min to complete. The link to the survey was also available as a bulletin posted on the intranet of Tafel Deutschland, which is accessible to all member food banks. The person responsible for the local food bank was requested to respond to the survey. Nine fuel vouchers (three of each, valued at €500, €300, and €100) were raffled among all participating food banks. By 23 October 2017, 281 of the 934 food banks had participated in the survey. In order to increase participation amongst food banks, a shorter version of the survey was developed and all food banks that had not yet participated received a reminder email containing the link to the shortened survey, which took approximately 30 min to complete. Additionally, food banks were contacted by telephone and encouraged to participate by answering the survey over the phone. This increased participation in the survey by another 273 additional food banks.

The study was approved by the Ethics Committee of the University of Hohenheim, Stuttgart.

### 2.1. Measures

The addresses of all food banks associated to Tafel Deutschland were received from Tafel Deutschland [26]. The most recent publicly available data on recipients of social welfare at district level is from the end of 2015 [27]. Publicly available shape files of German districts and district-free cities as well as of municipalities with the number of residents were retrieved from the Service Center of the Federal Government for Geo-Information and Geodesy [25].

The development of the survey questionnaire was guided by intense literature research [1,8,28,29] and through consultations with staff from Tafel Deutschland. It contained questions of the following domains: distribution schemes, services and projects of the food bank, food bank users, distributed food, food donors, food bank staff, and perceived challenges of the food bank in 2017. In addition, the long version of the questionnaire included questions on food bank’s facilities including storage space, waiting room(s) and transportation vehicles, organic waste accrued at the local food banks, and use of materials of the umbrella organization Tafel Deutschland. Due to reasons of clarity, these latter topics will not be included in the present analyses. Since the majority of the local food banks were not able to state the exact change in weight of food distributed or the number of users per month in 2017 compared to 2016, they were asked to rank possible changes from −2 (more than 20% less in 2017 compared to 2016), −1 (1–20% less), 0 (equal), +1 (1–20% more), +2 (more than 20% more) for both number of users and donated food weight.

The shortened questionnaire also covered all of the domains presented by this article, but in less detail (e.g., by asking for only the number of users rather than for the number of users and the number of visits). A selection of the questionnaire content is provided in the Supplementary File 1.

### 2.2. Geographical and Statistical Analyses

Addresses of all food banks were geocoded using MMQGIS [30] in the freely available GIS (geographic information system) application QGIS (version 2.18.16) [31].

Districts and municipalities with and without at least one food bank available were identified by using the point-in-polygon function in QGIS [31].

The coverage rate of the food banks was determined by calculating the number of districts and municipalities with at least one food bank located in them. Moreover, the number and proportion of residents and of residents receiving welfare benefits living in a district or district-free city with at least one food bank was calculated. Differences in the number of residents between municipalities with and without at least one food bank and differences in the number of residents, the number of welfare recipients, and the proportion of welfare recipients between districts with and without at least one food bank were tested by the *t*-test for independent samples.

Descriptive statistics (mean, standard deviation, median, sum, percentage) were calculated to illustrate the basic characteristics of the participating food banks, the food bank users as well as changes in the number of food bank users per month in 2017 compared to 2016, the food distributed as well as changes in the weight of food distributed per month in 2017 compared to 2016, the food donors, the food bank workers, and the challenges of the participating food banks.

Differences in the central tendencies of ordinal data or data not normally distributed were tested with the Mann–Whitney test for two groups; the Kruskal–Wallis test was used for more than two groups. Differences in continuous data were tested with *t*-tests for two groups.

Multivariate linear regression models were applied to identify resources of local food banks and the percentage of welfare recipients in the district the food bank was located in that predicted the number of users per month. Variables included in the regression analyses were the number of programs offered, the weight of food distributed per month, the number of workers, the number of services related to food, the number of services unrelated to food, the weight of food each user received per month, the percentage of volunteers of all workers, and the number of welfare recipients in percentage of the population in the district the food bank was located in. Backward selection based on Akaike information criterion was applied to receive a parsimonious model.

To examine the associations of both major challenges of participating food banks (lack of volunteers, lack of food) with the resources and demands of the food bank, logistic regressions were conducted. In the first logistic model, the variables of resources and demands in 2017 were included in the analyses. In the second logistic model, ranked possible changes in the number of users and the weight of food distributed in 2017 compared to in 2016 were included.

Since the number of users per month and the weight of food distributed per month were highly skewed, these variables were log-transformed before conducting regression analyses.

A *p*-value of <0.05 was considered to be significant. Data cleaning, preparation, and visualization were performed using Microsoft Excel 2007 (Microsoft Corporation, Redmond, WA, USA). Statistical analyses were performed using R, version 3.4.3 (R Foundation for Statistical Computing, Vienna, Austria) [32].

## 3. Results

At first, results of the geographic analyses will be shown before presenting the descriptive survey results including services provided by food banks, food bank users, food distributed by food banks, food bank workers, and challenges of food banks. Finally, results of multiple regression analyses will be shown.

### 3.1. Geographic Analyses

There was at least one food bank in 6.89% (*n* = 779) of all German municipalities in 2015, but 53.02% of residents lived in municipalities with at least one food bank. Municipalities with at least one food bank had a significantly larger number of residents (M = 55,935) than municipalities without a food bank ((M = 3667), t(778.23) = −8.4648, *p* < 0.0001). When considering the municipalities with at least 10,000 residents, which correspond to a so-called “big town” (“große Kleinstadt”) or larger, the percentage of municipalities with at least one food bank increased to 41.18% (*n* = 649).

At the next level of administrative units, 88.81% of districts had a least one food bank. The districts with at least one food bank had a larger number of residents (M = 214,983) than districts without a food bank ((M=120,592), t(280.58) = −5.9377, *p* < 0.0001). Districts with and without at least one food bank, however, did not differ in the number of welfare recipients as a percentage of the population (t(52.797) = −1.5547, *p* = 0.13). Overall, 93.40% of all residents and 94.52% of welfare recipients lived in districts in which they had access to at least one food bank.

**Figure 1:**
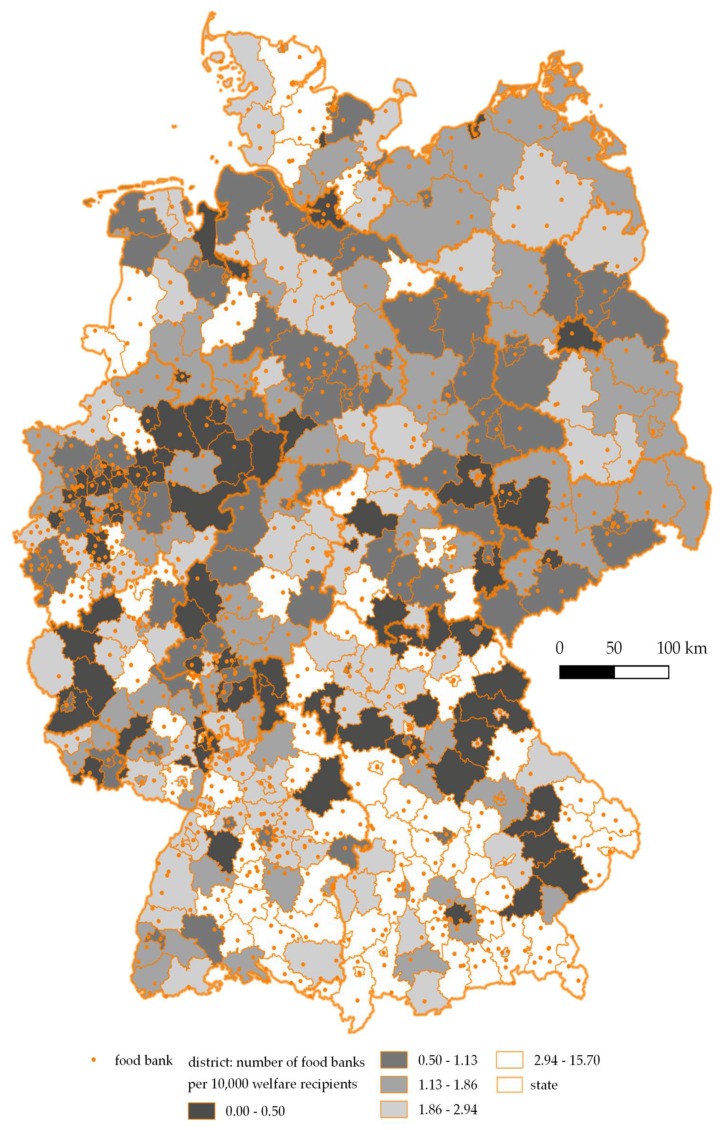
Number of food banks per 10,000 welfare recipients per district in Germany, 2014/2017.

As illustrated by Figure 1, the number of food banks per 10,000 welfare recipients was larger in districts of western Germany (M = 2.12) than in districts of eastern Germany ((M = 1.37), t(162.54) = 4.2424, *p* < 0.0001).

### 3.2. Survey

A total of 554 questionnaires—329 from the comprehensive online survey, 130 from the survey by phone, and 95 from the short online survey—were analyzed. Due to missing values and invalid data, fewer participating food banks were included in most of the further analyses. Food banks participating in the survey and those not participating in the survey did not differ in the type of community (χ(5) = 9.8542, *p* = 0.079), in the number of residents living in the district the food bank was located in (t(780.26) = −0.094, *p* = 0.93), or in the number of welfare recipients living in the district the food bank was located in (t(660.29) = −0.33, *p* = 0.74).

#### 3.2.1. Services Provided by Food Banks

The schemes for the distribution of the foods largely varied between participating food banks. The large majority of them distributed foods in more or less predetermined quantities based on household size for a small fee or at no cost at distribution points (84.85% of participating food banks). In contrast, food banks in the Southern state of Germany Baden-Württemberg (state-specific data not shown) tended to operate as “social supermarkets” where eligible individuals can purchase food at a greatly reduced price (18.25%). The difference between a distribution point and a “social supermarket” is that in the latter the clients pay for each food product they want to buy, whereas in a distribution point they pay a predetermined small fee. Whether clients are allowed to choose the food items they want differs between distribution points. On average, each food bank managed 2.21 (SD 3.0) distribution points and/or social supermarkets. Overall, 7.48% of the participating food banks delivered food to other organizations such as women’s shelters, youth centers, and drug rehabilitation facilities and served as so-called delivery food banks. In addition to these schemes, 10.40% of participating food banks also regularly supplied warm soups or other meals, whereas only a few of the participating food banks (3.28%) offer children a warm lunch at a so-called “Kinder Tafel” cafeteria. On average, each food bank managed 2.26 (SD 2.81) service programs (distribution points, social supermarkets, delivery food banks, soup kitchens, and/or children’s food banks).

The majority of the distribution points (75.50%) and delivery food banks (56.67%) allowed users to collect food once per week, whereas supermarket-like shops (37.14%), soup kitchens (71.15%), and children’s food banks (62.50%) tended to be open every day.

In addition to these standard schemes, 45% of participating food banks offered at least one additional service related to food, nutrition, or cooking such as a delivery service for home-dwelling elderly or disabled clients, offerings of coffee and cake during the hours of food distribution, and/or offerings of food recipes; 50% of them provided at least one additional service unrelated to food such as a thrift store, school supplies and toys, and/or social counseling.

#### 3.2.2. Food Bank Users

Descriptive statistics of food bank users are presented in Table 1. Initially, data of 415 food banks were available. Since data of 49 food banks were inconsistent (number of child recipients aged less than 18 years and of adult users did not equal the overall number of users), data of 366 food banks were included in the analyses. As indicated by the large standard deviations, very large variations in the number of users were observed between participating food banks.

There were no significant differences in the number of users between food banks located in western or eastern Germany (U = 5090, *p* = 0.82).

For 89 districts, all available food banks participated in the survey. On average, 179 (SD = 137) welfare recipients per 1000 welfare recipients and 17 (SD = 17) residents of 1000 residents used a food bank in the district.

For 152 food banks, data of user households by source of household income were available (Table 1).

**Figure 2:**
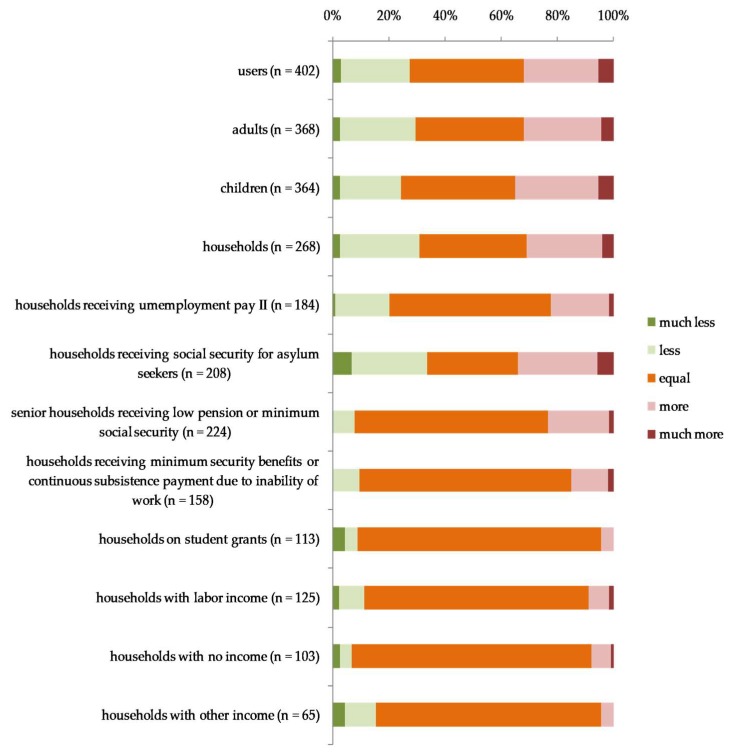
Comparison of the number of users of participating food banks per month in 2017 and 2016 in percent of participating food banks.

**Figure 3:**
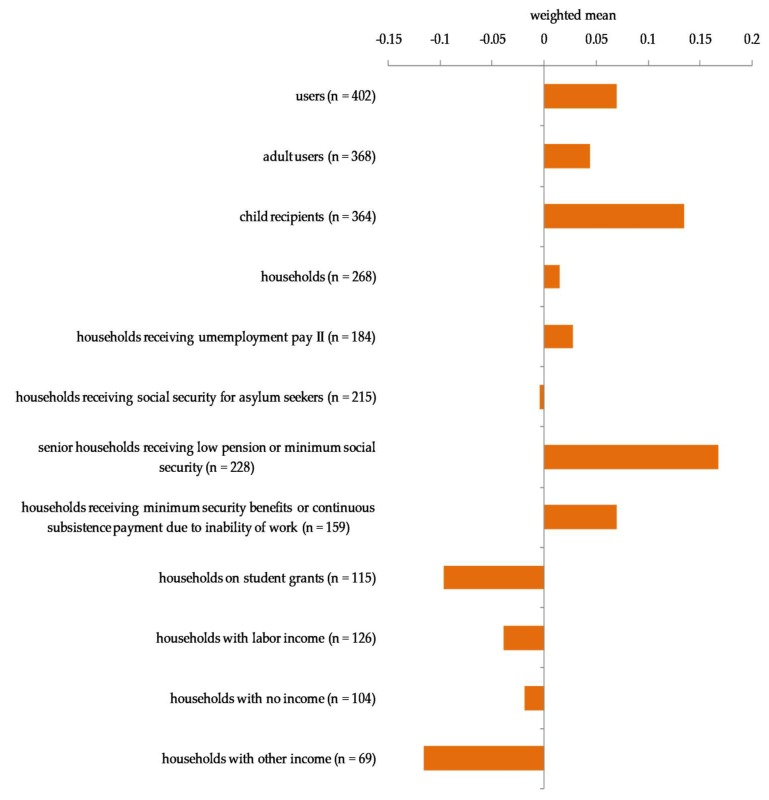
Number of users of participating food banks per month in 2017 compared with 2016 on a five-point scale.

As illustrated in Figure 2, more than half of the participating food banks reported that the number of users per month had increased in 2017 compared with in 2016. The weighted mean of reported scale points indicated an increase of the number of users per month in 2017 compared with 2016 (Figure 3). The ranked increase was higher among child recipients than among adult users, but the difference was not statistically significant (U = 62,648, *p* = 0.20).

Participating food banks reported most changes for households receiving support according to the Asylum Seekers Benefit Act (Figure 2). The Kruskal–Wallis test revealed that there was a significant difference in the ranks indicating changes in the number of households per month in 2017 compared with 2016 between the household groups by income (H(7) = 16.949, *p* = 0.018). A posthoc test using Mann–Whitney tests with Bonferroni correction showed that the ranks for households receiving a low retirement or minimum social security benefits for the elderly was significantly higher than the ranks for households receiving student grants (*p* = 0.007), for households with labor income (*p* = 0.022), and for households with other income (*p* = 0.022).

#### 3.2.3. Food Distributed by Food Banks

The mean weight of the food distributed monthly by each of the 328 food banks for which data were available amounted to 25.97 t (SD = 51.59). However, large variations could be observed and the distribution was highly skewed (median= 8.00 t). The mean weight of food per user per month was 23.92 kg (SD = 77.58 kg) and the median was 11.28 kg. There were no significant differences in the weight of the distributed food per month (U = 4647, *p* = 0.24) or in the weight of food per user per month between food banks in western and eastern Germany (U = 4547, *p* = 0.16).

**Figure 4:**
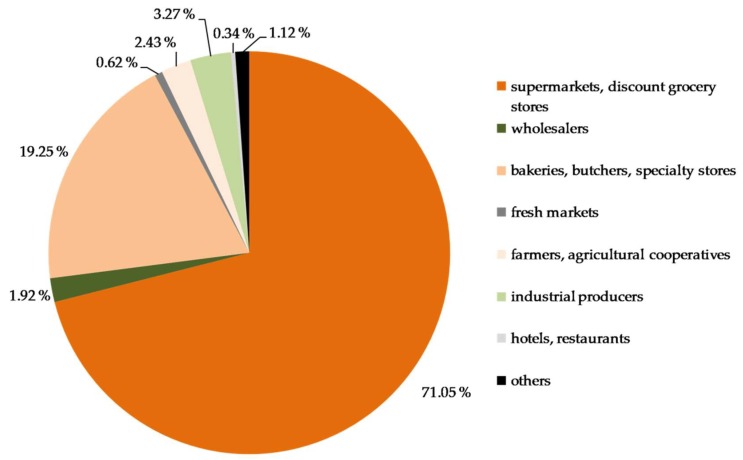
Types of regular food donors of participating food banks in percent, 2017.

The large majority of distributed food (82.29%) came from regular donors such as retailers. Less than 20% of distributed food came from single events or irregular donors (8.02%), the federal association Tafel Deutschland, state associations, and/or local distribution centers (7.68%), and/or from other sources (2.72%). Types of regular food donors are shown in Figure 4. Food banks reported receiving food from an average of 32.32 (SD = 34.25) regular donors.

**Figure 5:**
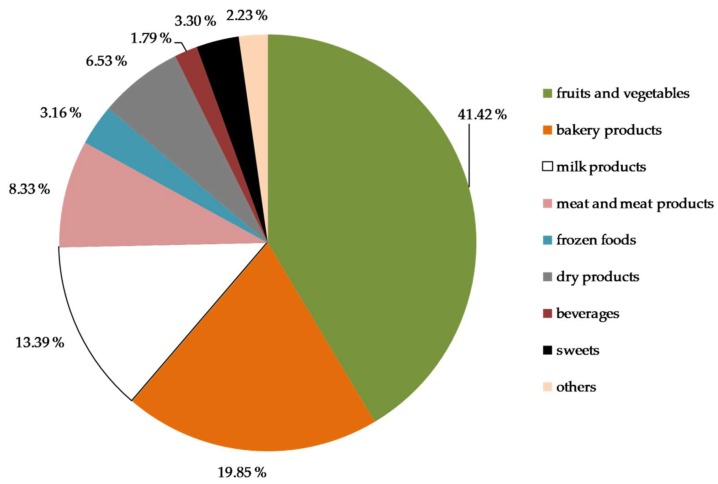
Categories of food distributed by participating food banks in percent, 2017.

**Figure 6:**
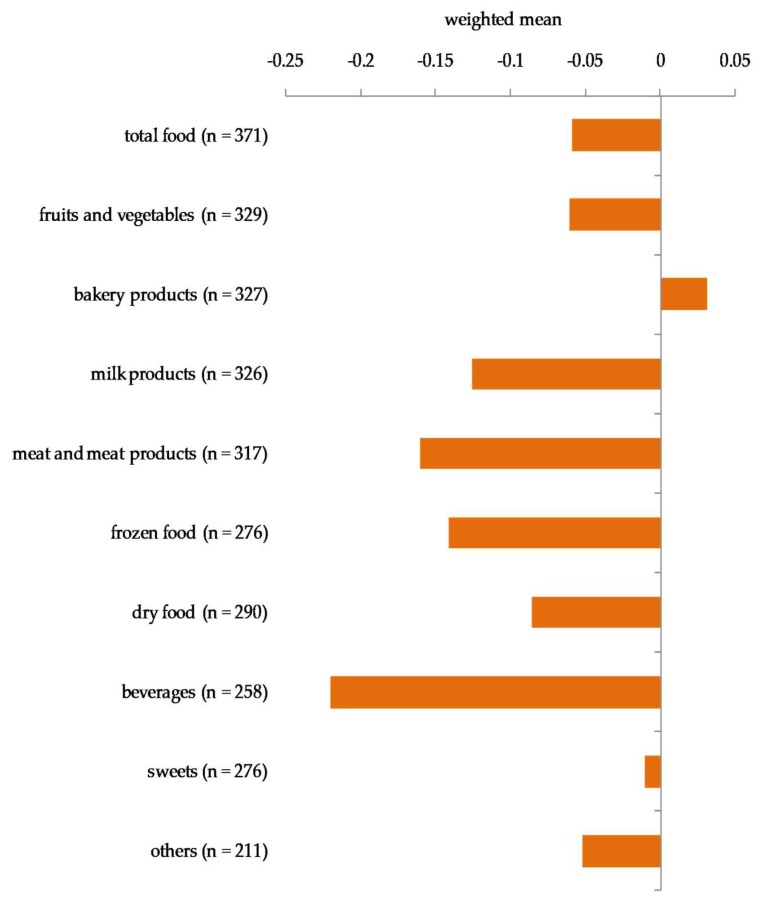
Weight of food distributed monthly by participating food banks in 2017 compared with2016 on a five-point scale.

As seen in Figure 5, the majority of food distributed per month was fruits and vegetables, followed by baked goods such as bread and pastries, milk products, and meat and meat products. Dry and frozen food, beverages, and sweets were distributed only in relatively small amounts. With the exception of baked goods, the amounts of almost all food groups were reported to have decreased in 2017 compared with 2016, as illustrated by Figure 6.

Overall, 47.45% of participating food banks reported that they infrequently (25.12%), sometimes (defined as once per four distribution days; 12.56%), often (defined as twice to thrice per four distribution days; 6.05%), or always (3.72%) had supply constraints, i.e., not enough food to cover demand in the months prior to the survey. Nearly 75% (74.51%) of them responded with a reduction in the amount of distributed food per household, 29.41% attempted to acquire more food from donors, 11.76% of them limited the membership and turned people seeking assistance away, and 7.48% implemented other measures to restrict access or to increase supply.

In contrast, 49.25% of participating food banks reported that they infrequently (32.84%), sometimes (11.94%), or always (4.48%) collected more food than they needed in the months before the survey. The majority of them (79.80%) distributed food they did not need to other nearby food banks, 51.52% of them froze or preserved food, 41.41% distributed excess food to other charitable organizations, 40.40% supplied users with more food, 13.13% threw excess food away, and 21.21% implemented other measures such as delivering the food to farmers for animal feed.

#### 3.2.4. Food Bank Workers

The large majority (89.97%) of people working in the 387 participating food banks for which data were available were volunteers. On average, every participating food bank had 59 (SD = 56) volunteers with large variations being observed. Volunteers were mostly 65 years or older (68.46% of volunteers) and female (61.52%).

Overall, 64.16% of the participating food banks had some paid staff, of which the mean number (M = 7, SD = 21) was much lower than that of volunteers. The majority of paid workers were participating in a government-subsidized employment scheme, the so-called One-Euro-Jobs (42.01% of paid workers). Only a few amongst the paid staff were permanent employees (0.67% of paid workers). The number of workers (M = 33.02, SD = 33.90) as well as the number of volunteers as a percent of the total number of workers (M = 65.69, SD = 28.47) were significantly lower for food banks located in eastern Germany than for those located in western Germany (number of workers: M = 82.00, SD = 74.01, t(183.51) = 6.96, *p* < 0.0001; number of volunteers in percent of total number of workers: M = 91.48, SD = 14.47, t(58.01) = 6.32, *p* < 0.0001).

Nearly 20% of all workers (volunteers and paid staff) were eligible to use a Tafel food bank and approximately 2% of all workers were refugees.

On average, volunteers worked 33.23 h (SD = 38.02) and paid workers worked 79.55 h (SD = 47.80) per month in a food bank with large variations observed among food banks.

#### 3.2.5. Challenges of Food Banks

Overall, 34.27% of the 321 participating food banks for which data were available stated that they had no challenges or problems in the last months. If problems were reported, the most frequent problem was a lack of volunteers (33.96% of participating food banks), in particular of volunteers with driver licenses who could pick up the food from retailers, followed by a lack of food (19.63%), in particular of milk products, meat, and sausages, and a lack of financial resources as well as lack of appropriate space (16.51% each).

#### 3.2.6. Associations

Results of multiple linear regression of the log-transformed number of Tafel users on predictors are shown in Table 2. The predictors accounted for 39.44% of the explained variance in the number of users.

The odds of having a lack of volunteers were significantly associated with working time per month per volunteer (b = 0.011, 95% CI 0.001, 0.020, OR 1.01, *p* = 0.026), but not with the log-transformed number of users per month, the weight of food distributed per month, or the number of volunteers in percent of the total number of workers. The model analyzing the association of a lack of volunteers and ranked possible changes in the weight of food distributed and the number of people served revealed that the odds of having a lack of volunteers decreased with an increase of food distributed per month in 2017 compared with 2016 (b = −0.57, 95% CI −1.04, −0.12, OR 0.57, *p* = 0.015).

The odds of having a lack of food was not significantly associated with the log-transformed number of users, the log-transformed weight of food distributed per month, the number of workers, the number of programs, or the number of food donors.

However, in the models analyzing the association of a lack of food and ranked possible changes in the weight of food per month and in the number of users per month in 2017 compared with 2016, the odds of having a lack of food significantly increased with a decrease of food per month in 2017 compared with in 2016 (Table 3).

## 4. Discussion

This study revealed that a Tafel food bank was in operation in more than every second “big town” and nearly all residents and welfare recipients had access to at least one food bank in the district they lived in. Thus, Tafel Deutschland appears to provide a comprehensive net of local food banks throughout the country. In addition to the regular supply of mainly fresh produce, many food banks provided additional services such as social counseling and meal recipes, which may directly or indirectly impact users’ food security. However, the density of food banks per 10,000 welfare recipients differed between parts of former East and West Germany with a lower density in eastern parts.

An analysis of the roots of this pattern is beyond the scope of this paper, but an explanation might be that the total number of workers as well as the number of volunteers as a percentage of all workers was significantly lower among participating food banks located in eastern compared with western Germany. Differences in volunteer engagement between eastern and western Germany were also observed in the German representative volunteer survey of 2014 and have been explained by the long history of Germany’s separation, differences in unemployment rate, economic performance, and demographic change [33]. Given that food banks’ assistance was largely based on volunteer labor, the number of available volunteers is a main pillar in the establishment of a food bank. The odds of reporting a lack of volunteers increased with increasing working time per volunteer, indicating that the workload of volunteers rather than the sole number of volunteers seems to be one of the limiting factors in balancing the supply and demand of existing food banks.

The volunteer-driven nature of the German Tafel is similar to food banks in other high-income countries such as Canada [28], the USA [1], and Spain [5]. In contrast to food banks in these countries, the German food banks neither involve the public sector nor receive food or other subsidies from the European Union or other national or international political organizations. German food banks heavily rely on surplus food donated from retailers and bakeries, whereas goods from producers or other wholesale donors constitute only a small part of the overall amount of food. This system shapes the quantity, quality, and reliability of the food to be distributed. On one side, it allows local food banks to supply fresh food such as fruits and vegetables, which are food products that food-insecure people tend to consume in particularly low amounts [29], although its health impacts are well known [34]. Moreover, it helps to prevent food being thrown away. According to a study under the authority of the Federal Ministry of Food and Agriculture from 2012, food waste from retailers accounts for 490,000 tons per year, of which around 38% are donated to charitable organizations such as Tafel food banks [35]. On the other side, the dependency of local food banks on donations of surplus food by local retailers makes the quantity, variety, and quality of available food highly unpredictable. Variations have also been observed in the nutritional quality of food distributed by food banks in other high-income countries even if they received government funding, such as U.S. food banks for The Emergency Food Assistance Program [36], but they tend to provide less fresh food [8]. Although this study is not able to evaluate the food donation systems of food banks in other high-income countries, it seems that donations from government programs might not necessarily make the amount of food more predictable.

Food banks participating in the survey reported not only a temporary lack of food but also an irregular surplus of food. More than 40% of food banks that reported this occasional surplus passed this food on to its users even if the amount was likely more than the user household could consume. Although the types of surplus food were unknown and it remained unclear whether users consumed this food or shared it with neighbors or friends, this practice forced the users to solve the problem and potentially might have unfavorable impacts on users’ diet and health, e.g., if the surplus food consists of bread and pastries. This holds particular truth given that the association between food insecurity and obesity, the food insecurity–obesity paradox, is well known [37,38]. A diet heavily reliant on food bank types of food may exacerbate existing chronic conditions such as diabetes [39]. Most of the research on the relationship between food insecurity and obesity has, however, been conducted in the USA, where a so-called monthly food stamp cycle was identified [40]. At least among subgroups of recipients of the Supplemental Nutrition Assistance Program or residents with low income, food intake and food expenditures were shown to dramatically increase after food assistance [40,41] or after transferred income [42] and then to decrease over time before the next assistance/income. A similar monthly variability has also been observed in the use of soup kitchens [43]. Tafel users tend to visit the food bank every week [15] and this study is not able to reveal whether similar weekly cycles also exist among food bank users in Germany, but an infrequent oversupply of food approaching its best-before date for people already at high risk of being overweight or obese and food insecure [6,15] may be contraproductive and could unintentionally support periods of overeating.

Given that the odds of reporting a lack of food were neither related to the weight of total food distributed per month nor to the weight of food a user received per month, but to a decrease in the total weight of food distributed per month in 2017 compared with 2016, it appears that participating food banks tended to evaluate the quantity of the available food based on their experiences rather than on objective measures. This might contain the risk of dramatic miscalculation. Therefore, food banks and their users might benefit from reliable, user-friendly tools to assess the quantity and quality of the food distributed and from national guidelines regarding the amount and quality of distributed food. Food banks in other countries such as the USA have already applied diverse instruments to assess the nutritional quality of distributed foods by, e.g., nutrition profiling [44,45,46]. The impact of the implementation of such tools depends, however, on the willingness of food bank managers and of food donors to accept restrictions in the quantity and quality of food and on the limited personnel capacity of the food banks.

Just recently, public and political debate about the role of the Tafel food banks in the German welfare system has increased again [47]. Similar to other European countries [11,48,49], the Tafel movement is considered a seismograph for social developments [50,51] and changes in the number of food banks or its users have been interpreted to indicate changes in the food security rate [52]. The results of this study challenge these interpretations. Most user households relied on public welfare, but only a small part of eligible welfare recipients used a food bank. In 79 districts for which all available food banks participated in the survey, on average, 179 welfare recipients per 1000 welfare recipients and 17 residents per 1000 residents used a food bank. These numbers of usage were larger than the numbers revealed by a study in Berlin [9], but much lower than the prevalence rate of food insecurity of 4.3% (i.e., 43 per 1000 residents; margin of error at 90% confidence ±1.44%) reported by the Food and Agriculture Organization of the United Nations for Germany [17]. Thus, the majority of food-insecure individuals do not appear to use a food bank. One of the possible manifold reasons for this mismatch might be that more than every tenth food bank participating in the survey reported limiting access to its assistance due to a lack of food. In addition, as reported by Tafel users as well as food bank workers, shame and fear of stigmatization associated with food bank use [53,54] might potentially prevent food-insecure people from seeking a food bank’s assistance. Although motives for not using a food bank were not assessed by this study, participants reported that shame was a significant barrier, in particular among older people, to seeking assistance from a food bank. Furthermore, compared with other high-income countries, grocery prices in Germany are among the lowest, with budget supermarkets significantly undercutting other chains and driving down prices [55].

Nevertheless, a previous study showed that the distribution of food pantries mirrored the distribution of welfare recipients in Berlin [9], and the present study revealed that the number of food bank users was at least partly a function of the percentage of welfare recipients in the district the food bank was located in. Among all user household groups, user households receiving a low retirement or minimum social security benefits for senior citizens increased highest from 2016 to 2017. Actually, in Germany the rate of older people being at risk of falling into poverty has steadily increased over the last few years [56], whereas the unemployment rate has decreased [57].

### Limitations

One of the major limitations of this study is the limited reliability of participants’ responses. All data collected by the survey relied on self-reports. Given that food banks focus on the distribution of food and are driven by volunteers, some food banks were not able to provide detailed records, for instance, of the weight of food or the number of users. Additionally, there are no national standard procedures of data collection, and the data presented here might be subject to estimation errors.

Potential changes in the number of users and the weight of distributed food per month were retrospectively requested, which increases the risk of memory bias. The cross-sectional design of the study precludes the drawing of causal relationships.

Given that food banks participating and those not participating in the survey did not differ in location characteristics, it can be assumed that the results are representative for all food banks in the federal umbrella organization Tafel Deutschland. Due to the heterogeneity of food banks in many other characteristics, however, some uncertainty about the representativeness of any food bank sample remains.

Results of the additional services provided by the participating food banks might also give an incomplete picture of services offered in the context of the Tafel, since a service was only recorded if it was administered by the participating Tafel itself. However, there were services located in the same facility as the food bank but being provided by other organizations, which were not recorded. 

Lastly, the latest data of the number of welfare recipients per district were available from 2015, whereas the data collected by the survey were from 2017. Although changes in the number of welfare recipients as a percent of the population were presumably small [58], the differences in the years the data were collected should be considered carefully when interpreting the results.

## 5. Conclusions

The German Tafel system provides a wide range of food assistance schemes supplying food of high nutritional value and additional services with the potential to impact individuals’ diet and food insecurity. It appears that changes in the number of food bank users and their source of income partly mirror changes in the at-risk-of-poverty rate and social welfare in Germany, but there obviously are unknown factors influencing the usage of food banks. The number of food bank users seems to be an inappropriate indicator of the food insecurity rate, which can be taken as a sign of the need for implementing a regular food security monitoring system.

Due to the dependency of food banks on volunteers and food donations, they are hardly a reliable food source for parts of the population who are vulnerable to food insecurity due to their socio-economically disadvantaged situation. The obvious strain between the reliance on food donations and the response to the shifting needs of food bank users entails the risk of volunteer overload and inappropriate short-term solutions such as providing users more food than needed. One solution could be collaborations with dieticians and other public health and nutrition professionals to receive support regarding the dietary needs of food bank users. However, this will only be effective if food bank users are able to use the food bank to supplement their usual diet (as is the claim of Tafel Deutschland) rather than to rely on food banks as their primary or even only source of food. 

To understand contributing factors as to which individuals use a food bank and why, further research is needed. Moreover, the impact of a food bank’s food assistance on an individual’s diet and food security level needs to be investigated.

In general, food banks’ growth and assistance should be accompanied by vigilant coalitions of the charitable food organizations, the social sector, and professionals of social, nutritional, and health sciences in order to have a working system that supports those in need and contributes to the reduction of food waste.

## Figures and Tables

**Table 1:** Sociodemographic characteristics of food bank users in Germany, 2017.

**Characteristics of Food Bank Users**	**Mean**	**Standard Deviation**	**Median**	**% of Users**
Users	1559	3126	726	100
Adult users	1120	2684	480	72
Child recipients	440	724	209	28
Households	696	952	300	/
**Characteristics of Households of Food Bank Users**	**Mean**	**Standard Deviation**	**Median**	**% of Households ^2^**
Households receiving unemployment pay II ^1^	260	436	115	49
Households receiving social security for asylum seekers	139	255	80	26
Senior households receiving low pension or minimum social security	83	131	40	16
Households receiving minimum security or disability benefits	37	63	18	7
Households with low labor income	10	25	0	2
Households with other income/no income	6	19	0	1

Note: For the characteristics of food bank users, data of 366 participating food banks were available and for the characteristics of households of food bank users, data of 152 participating food banks were available; excl. individuals who receive food from other noncharitable organizations such as women’s shelters, schools, youth clubs, etc. that are delivered by delivery food banks; ^1^ unemployment pay II is a basic security benefit for job-seekers; ^2^ in rounded percent of the overall sum of households for which the source of household income was available.

**Table 2:** Association between the log-transformed number of food bank users and food bank resources and district character. Results of multiple linear regression analyses.

	β	*p* Value	95% CI
Intercept	5.23	<0.0001	4.93, 5.53
Number of service programs ^a^	0.044	0.05	−0.0007, 0.090
Weight of distributed food per month ^b^	0.197	<0.0001	0.13, 0.26
Number of workers ^c^	0.005	<0.0001	0.003, 0.006
Number of additional services unrelated to food	0.065	0.13	−0.020, 0.15
Number of welfare recipients in percent of the population	0.070	<0.0001	0.038, 0.010

^a^ including distribution points, social supermarkets, delivery food banks, soup kitchens, and children’s food banks; ^b^ log-transformed; ^c^ including volunteers and paid workers; β: unstandardized regression coefficient; CI: confidence interval.

**Table 3:** Association between the log of having a lack of food and the ranked possible changes in the number of users and the distributed food. Results of logistic regression analyses.

	β	*p* Value	95% CI	OR
Intercept	−1.82	<0.0001	−2.27, −1.42	0.16
Ranged possible changes in the weight of food per month in 2017 compared with 2016	−1.16	0.0001	−1.78, −0.60	0.31
Ranged possible changes in the number of users per month in 2017 compared with 2016	0.31	0.16	−0.12, 0.76	1.37

β: unstandardized regression coefficient; CI: confidence interval; OR: odds ratio.

## Supplementary Materials

The following are available online at http://www.mdpi.com/1660-4601/15/7/1485/s1, Table S1: Questionnaire content, developed for a representative survey among German food banks: questions, answer options, variables (selection).
