# Supplementary material for: The German Food Bank System and Its Users—A Cross-Sectional Study
Source: Int J Environ Res Public Health. 2018 Jul 13;15(7):1485. doi: 10.3390/ijerph15071485 (PMC6069247; doi:10.3390/ijerph15071485)
Supplement: Supplementary file 1 [file ijerph-15-01485-s001.pdf]

Article, supplementary material

The German food bank system and its users – a cross-sectional study

Anja Simmet[1]*, Peter Tinnemann[2]andNanette Stroebele-Benschop[1]

[1] Institute of Nutritional Medicine, University of Hohenheim, Stuttgart, 70593, Germany; Anja.Simmet@uni-hohenheim.com; N.Stroebele@uni-hohenheim.de
[2] Institute for Social Medicine, Epidemiology, and Health Economics at the Charité University Medical Center, Berlin, 10117; peter.tinnemann@charite.de
* Correspondence: Anja.Simmet@uni-hohenheim.com; Tel.: +49 176 22 88 9784

Table S1. Questionnaire content, developed for a representative survey among German food banks: questions, answer options, variables (selection).

Questions	Definitions / Answer options	Variables
Which standard programs does your food bank provide regularly? (several answers possible)	Distribution point: The clients of your food bank are regularly allowed to receive more or less predetermined food parcels for a low fee or at no costs.<br>Social supermarket: The clients of your food bank are regularly allowed to choose the food items they want and pay for each food item a low price.<br>Delivery food bank: Your food bank regularly delivers food to other social organizations or other social organizations can pick up the food from your food bank.Please do not mistake with delivery service for old or disabled clients!<br>Soup kitchen: Your food bank regularly provides warm meals for a small fee or at no costs.<br>Children food bank: Your food bank regularly provides (warm) meals to children.<br>Others (please specify)	Number of food banks with distribution points / social supermarkets / etc. in percent of participating food banks
Please indicatethe number of distribution points of your food bank.	open	number (mean / standard deviation) of distribution points
Please indicatethe number of social supermarkets of your food bank.	open	number (mean / standard deviation) of social supermarkets
How often are clients allowed to receive food from distribution point(s) / to shop in the social supermarket(s) / be delivered from the delivery food bank	As often as they want / every day<br>Twice per week	Allowed usage frequency in percent of food banks with distribution points

/ to receive a warm mealfrom the soup kitchen / to receive a mealfrom the children food bank / to receive food or a mealfrom other programs of your food bank?	Once per week<br>Every other week<br>Once per month<br>Others (please specify)	/ with social supermarkets…
Which additional services related to food does your food bank provide? (several answers possible)	Delivery service: Your food bank delivers food to home dwelling elderly or disabled clients.<br>Providing coffee / cake / snacks to clients during food distribution: Your food bank provides coffee, cake and/or snacks to clients for a small fee or at no cost during food distribution.<br>Offering recipes: Your food banks offers recipes for food items your food bank provides.<br>Cooking courses for adults: Your food bank provides cooking courses for adults.<br>Cooking courses for children: Your food bank provides cooking courses for children.<br>Nutrition education for adults: Your food bank provides nutrition education for adults.<br>Nutrition education for children: Your food bank provides nutrition education for children.<br>No food related service<br>Others (please specify)<br>Definition of users: all people who receive food or meals from your food bank incl. household members.<br>Please note that users will be not counted more than once, even if they may visit your food bank more than once per month.	Number of food banks providing at least one additional food related service in percent of participating food banks.<br>Number of food banks providing food related service xy in percent of participating food banks.
Please indicatethe number of users per month in 2017.	open	Number of users per month in 2017
Please indicatethe number of adult users per month in 2017.	open	Number of adults users per month in 2017
Please indicatethe number of child recipients per month in 2017.	open	Number of child recipients per month in 2017
